# The foot-related clinical characteristics of people with diabetes in an Australian regional setting

**DOI:** 10.1186/1757-1146-4-S1-P44

**Published:** 2011-05-20

**Authors:** Byron Perrin, Marcus Gardner, Susan Kennett

**Affiliations:** 1 La Trobe Rural Health School, La Trobe University, Bendigo, Victoria, 3550, Australia; 2 Musculoskeletal Research Unit, La Trobe University, Bundoora, Victoria, 3086, Australia; 3 Bendigo Health, Bendigo, Victoria, 3550, Australia; 4 Bendigo Community Health Services, Bendigo, Victoria, 3550, Australia

## Background

The podiatrists of the regional Victorian health organisations Bendigo Health (BH) and Bendigo Community Health Services (BCHS) recently evaluated a collaborative podiatric model of care. This paper describes the clinical and demographic characteristics of the patients seen within this model over a three-month period.

## Methods

A three-month prospective clinical audit of the podiatry services involved in the podiatric model of care was undertaken. Basic demographic variables were recorded in addition to the UT risk classification at baseline and incidence of ulceration. Statistical analysis was undertaken to explore the differences in the demographic variables with respect to both risk category at baseline and incidence of ulceration.

## Results

576 patients were seen during the three-month period. Mean age was 71.3 + 11.6 years, with males accounting for 53.3% of the cohort. The majority (95.8%) of the sample had type 2 diabetes and the mean duration of diabetes was 12.08 + 10.0 years. At baseline, 51.4% of the cohort was classified as having “no neuropathy” and 10.6% classified as having an “active pathology”. Those at higher risk of future foot pathology at baseline were younger (F= 11.9, p<0.0005) and had a longer duration of diabetes (F= 31.7, p<0.0005). Males were associated with higher risk (χ^2^= 40.3, p<0.0005).Thirty-six (6.3%) people developed incident diabetes-related foot ulceration. Those that developed ulceration were younger (t= 3.5, p= 0.001) and had a longer duration of diabetes (t= -3.3, p= 0.002). The proportion of people with type 1 diabetes who developed incident ulceration was higher than for people with type 2 diabetes (χ^2^= 9.1, p=0.003).

## Conclusions

The foot health of this large cohort ranges across the entire spectrum of diabetes-related foot complications. There are consistencies with other relevant populations with respect to age, gender, diabetes type and the proportion of males and adults with type 1 diabetes at higher risk of future foot pathology. Although it is unusual to see those at higher risk of problems being a younger age, this may be offset by a longer duration of diabetes. The three-month incidence of ulceration was high, reflecting the clinical nature of the cohort.

